# Updated Mineral Composition and Potential Therapeutic Properties of Different Varieties of Olive Leaves from *Olea europaea*

**DOI:** 10.3390/plants12040916

**Published:** 2023-02-17

**Authors:** Natália M. de Oliveira, Lara Lopes, Maria Helena Chéu, Eugénio Soares, Diana Meireles, Jorge Machado

**Affiliations:** 1 ICBAS-UP Laboratory of Applied Physiology, Institute of Biomedical Sciences Abel Salazar, University of Porto, 4050-313 Porto, Portugal; 2 CBSin, Centre of Biosciences in Integrative Health, 4250-105 Porto, Portugal; 3 RECI—Research Unit in Education and Community Intervention, Instituto Piaget—ISEIT/Viseu, 3515-776 Viseu, Portugal; 4 Laboratório Central de Análises, Universidade de Aveiro-UA, 3810-193 Aveiro, Portugal

**Keywords:** *Olea europaea* L. *folium*, inorganic composition, nutraceutical potential, intelligent bioactive compounds

## Abstract

*Olea europaea* L. *folium* has been studied for its potential nutraceutical properties. Quantitative and qualitative analyses were conducted on samples of Madural, Verdeal, and Cobrançosa elementary leaves and leave sprouts (*mamões*) collected in the region of Valpaços, Portugal. Mineral analysis determined the measurements of the levels of several macro- and micro-elements based on ICP-MS techniques. The inorganic analysis in this work allowed us to propose olive leaf extract (OLE) from different cultivars as a viable and affordable source of mineral substrates to address disorders related to essential elements such as Na, K, Mg, Ca, Mn, Fe, and Cu deficiencies. Given the importance of the research on novel therapies, finding a suitable substrate for extracting quality amounts of mineral is a priority. The physiological influence of enzymes dependent on minerals with regard to neuroinflammatory and neurobehavioral, metabolic, cardiovascular, osteodegenerative, anti-aging, pulmonary, and immunological defense disorders might dictate the importance of further research for designing supplementation based on the nutraceutical potential of OLE of these cultivars predominant in the northern region of Portugal.

## 1. Introduction

Olive tree byproducts such as the leaves have a compelling record of nutritional value and medicinal use [1]. The main reason for the cultivation of *O. europaea* L. has always been the production of olive oil [2], but the extraction of other byproducts such as olive leaves (*O. europaea* L. *folium*) is currently pursued for their therapeutic value, biological properties, and organoleptic characteristics [3]. The efficacy and specificity of the therapeutic responses of olive leaves can also be influenced by their mineral composition. In general, Na, Mg, P, K, Ca, Mn, Fe, Cu, Zn, and Pb contents are possible to find and are usually measured the most in olive leaves [4,5]. However, several additional mineral elements, even under residual levels, can be found and constitute a particular interest for evaluating eventual specific conditions in complementary therapy, namely Li, Si, B, Al, Cr, Co, Ni, As, Se, Rb, Sr, Mo, Cd, Sn, Sb, and Ba [6,7]. As is naturally expected, the mineral composition of leaves varies according to the soil and climate where the tree was planted. Studies in Greece and Portugal concerning the elemental composition of leaves, nutrient uptake, and usability by different varieties showed significant differences of the leaves even when they were grown under the same ecological conditions [8,9,10]. This may express specific metabolic answers for the macro- or micronutrient requirement in different olive cultivars, even when facing similar ecological factors [11,12]. However, specific metabolic dependence on soil mineral composition is possible to find in olive leaves; it is likely that the concentration of Ca is positively correlated with Mg and Na concentrations while being negatively affected by Fe uptake [4,10]. Usually, Ca, Mg, and Na ions in exchange sites are common cations of arid region soils. On the other hand, a strong positive correlation was observed between K and Fe uptake. Potassium fertilization is often practiced stimulating Fe uptake [13]. Moreover, Ca uptake negatively affected both N and K concentrations in the leaves, in addition to Fe [4,14,15]. Zinc concentration showed significant correlation with the N, K, and Mn concentrations of the leaves, while Ca, Cu, and Na uptake exhibited a significant negative correlation with N concentration [16]. All this rich potentiality of olive leaf extract (OLE) hangs on the quantity of molecule/compound that is absorbed and metabolized in an organism after ingestion, in which the latest progress in biotechnology allows to understand and find ways to create supplementation that potentiates the maximal absorption of those bioactives such as the design of ion-nanoparticles (ion-NPs) [17,18]. For this reason, it is crucial to understand the biochemical composition of olive tree leaves from the most relevant cultivars. Therefore, in this context, previous research was performed to assess the organic composition [19]. The present work aims to assess the inorganic parameters of the elementary leaves and “*mamões*” (leaf sprouts) of cultivars for which only data regarding the organic profile have been reported (data not shown). In this case, for their prominence in our culture and economy, we selected the three most characteristic olive cultivars from Trás-os-Montes, in the municipality of Valpaços, in the Vila Real district: Madural, Cobrançosa, and Verdeal Transmontana. The relevance of including the study of leaf sprouts (*mamões*) properties lies in the fact that these parts of the olive tree are often discarded by the producers for overtaxing nutrients and energy from the central axis of the plant, originating unusable material often taken for ruminant animal feed but mostly ends up being combusted or disposed of in sanitary landfills. An alternative for upcycling instead of the usual treatment as a waste byproduct potentially brings economic and environmental benefits, apart from relieving the burden of producers concerning removal and disposal costs.

## 2. Results

A mineral assessment of elementary leaf and leaf sprouts was made by screening the presence of 26 different elements. It was determined that Madural’s elementary leaf had a mineral content with a predominance in waning sequence as follows: K (11,534 mg), Ca, P, Mg, Si, Rb, Zn, Na, Mn, Sr, and Fe (31 mg) (Table 1). Verdeal’s elementary leaf was found to have a mineral predominance in the waning order of Ca (15,291 mg), K, P, Mg, Si, Rb, Al, Mn, Sr, Na, Fe, and Zn (21 mg) (Table 1). Finally, Cobrançosa’s elementary leaf showed predominance also in a waning order as follows: Ca (11,542 mg), K, P, Mg, Si, Na, Rb, Al, Sr, Mn, Ba, and Zn (15 mg) (Table 1). From Table 1, it is then noticeable that the major mineral components present in the elementary leaves of these cultivars are calcium (Ca), potassium (K), phosphorus (P), magnesium (Mg), and silicon (Si); Ca occupies the higher ranking for Verdeal and Cobrançosa, whereas K comes first in Madural.

In the analysis of leaf sprouts, we found that Madural’s cultivar has a predominance in the following waning order: K (12,892 mg), Ca, P, Mg, Rb, Si, Na, Sr, Al, Mn, B, Fe, and Zn (20 mg) (Table 1). Verdeal’s leaf sprouts showed predominance in the waning order of K (13,122 mg), Ca, P, Mg, Rb, Si, Na, Al, Mn, Zn, Fe, B, and Cu (11 mg) (Table 1). Finally, Cobrançosa’s leaf sprouts showed a mineral predominance of K (9614 mg), Ca, P, Mg, Si, Na, Rb, Al, Sr, Mn, Fe, Zn, B, Ba, and Cu (9 mg) (Table 1). Very much alike to the elementary leaves of Madural’s cultivar, the leaf sprouts of all three cultivars were determined to have a higher content of K followed by Ca, P, and Mg. The fifth most predominant element was Si in all cultivars’ elementary leaves and for Cobrançosa’s leaf sprouts, while in the leaf sprouts of Madural and Verdeal, the top fifth position was occupied by Rb.

## 3. Discussion

Screening the presence of 26 different elements, it was determined that the major mineral components present in these cultivars are calcium (Ca), potassium (K), phosphorus (P), magnesium (Mg), and silicon (Si). Ca occupies a higher ranking in Verdeal and Cobrançosa, whereas K comes first in Madural. Very much alike Madural’s elementary leaves, the leaf sprouts of all three cultivars were determined to have a higher content of K followed by Ca, P, and Mg. Fundamentally, the results of the inorganic analysis showed a composition in kg of leaves close to or above the DRI values for the following components: Ca, Cu, Fe, K, Mg, Mn, P, Sb, Sr (except for Verdeal’s leaf sprouts), and Zn. Among those metals currently considered essential for normal biological functioning, we found Na, K, Mg, and Ca and block transition metal elements Mn, Fe, and Cu [19].

Chronic hypocalcemia may be of multifactorial origin with osteoporosis being the most known issue and is usually prevented through a balanced diet and calcium supplements, sometimes associated with vitamin D and/or Mg supplementation [15,20,21]. A prosperous intake of proteins, Ca, and vitamin D demands higher levels of Mg, the same way that hypomagnesemia can lead to hypocalcemia and hypokalemia [22,23,24,25,26]. Malnourishment, obesity, and alcohol withdrawal might implicate deficiencies in inorganic phosphate (Pi) or adenosine triphosphate (ATP) related to bone health, myopathies, and central and peripheral nervous system and whose treatment calls for the proper regulation of phosphorus levels [27,28,29,30,31,32,33]. Manganese, an essential trace mineral, is involved in physiological processes as follows: (1) blood clotting and hemostasis in conjunction with vitamin K; (2) as a cofactor for several enzymes involved in the metabolism of different nutrients such as manganese superoxide dismutase, arginase, and pyruvate carboxylase reactive oxygen species (ROS) scavenging; (3) osteogenesis; (4) reproduction; (5) immune response; and (6) digestion [34]. Manganese deficiencies rarely happen except in children on long-term parenteral nutrition or with patients who have mutations in the metal transporter SLC39A8 gene. A restricted body of evidence shows that manganese deficiency in humans might be related to (1) impaired growth and bone formation in children, (2) skin rashes, (3) hair depigmentation, (4) diminished serum cholesterol, (5) changes in lipid and carbohydrate metabolisms, (6) changes in tolerance to glucose, (7) increased activity of alkaline phosphatase in men, and (8) mood changes or premenstrual pain in women [35]. Although Mn deficiency is rare to find, a concern for low Mn-dependent superoxide dismutase activity may be associated with cancer susceptibility. The increasing popularity of vegetarian lifestyle helps to prevent Mn deficiency; however, some vegetarian individuals are prone to iron deficiency [35]. Fe deficiency precedes anemia and several deleterious systemic effects because, when Fe levels are low, copper is absorbed mainly through the DMT1, and the role of copper in iron deficiency is to stimulate iron mobilization from stores by ceruloplasmin synthesis. When iron is abundant, such as in hemochromatosis, ceruloplasmin levels are low and associated with copper deficiency, which may be of importance for certain groups of patients receiving iron treatment. Copper is mostly found in the human brain, and its homeodynamics is balanced by absorption from the intestinal tract and by excretion in the bile. In addition, Cu/Zn superoxide dismutase 1 (SOD1) uses a cupric ion to catalyze the disproportionation of superoxide within the cytoplasm and therefore mitigate oxidative damage outside of the mitochondria [36]. Not only enzymes such as SOD1, and transcription factors are crucial for cell integrity, but also mechanisms related to copper have been shown to be a prospective therapeutic target for influenza A, lung inflammation, cancer, Alzheimer’s disease, Parkinson’s disease, Menkes disease, and Wilson’s disease, as well as obesity and nonalcoholic fatty liver disease (NAFLD) [37,38]. Understanding the metabolism of these ions aids in the advancement of treatment and diagnosis with nanoparticles (NPs) that can be used to regulate the levels of ions already present in organisms or, when produced as ion-NPs, to take advantage of homeostatic pathways present in the human body. NPs are generated by physicochemical processes whether through heat, ultrasound, or microwaves, and, when arranged in more complex structures, NPs acquire higher bioavailability, targeting capabilities and theragnostic properties [18]. As an example, changes occur in the taste of food when using Fe for food fortification, and side effects have been identified in oral iron medication such as nausea, vomiting, and diarrhea. For this reason, NPs might be a great solution to recreate a novel therapy targeting mineral and vitamin disorders. Given the importance of the research on these novel therapies, finding a suitable substrate for extracting quality amounts of mineral is a priority. The inorganic analysis in this work allowed us to propose OLE from different cultivars as a viable and affordable source of mineral substrates to address disorders related to essential elements such as Na, K, Mg, Ca, Mn, Fe, and Cu deficiencies. In particular, we suggest Cobrançosa’s leaf sprouts to address hyponatremia, Verdeal’s and Madural’s leaf sprouts for hypokalemia, Verdeal’s elementary leaves and Madural’s leaf sprouts for hypomagnesemia, Cobrançosa’s and Verdeal’s elementary leaves for hypocalcemia, Verdeal’s elementary leaves for Mn deficiency or Madural’s elementary leaves to avoid Mn excess, and Verdeal’s leaf sprouts for hypocupremia; finally, the elementary leaves of the three cultivars can be used to address sideropenia.

## 4. Materials and Methods

### 4.1. Chemical Reagents

Absolute ethanol was obtained from Fisher Chemical (Loughborough, UK). Methanol, gallic acid, Folin–Ciocalteu reagent, sodium carbonate (Na_2_CO_3_), boron trifluoride (BF_3_), and 1,4-dioxane were purchased from Sigma (St. Louis, MO, USA). Nitric acid (HNO_3_), hydrogen peroxide (H_2_O_2_), Kjeldahl tablet catalyst, sulfuric acid, boric acid, potassium hydroxide (KOH), anhydrous sodium sulfate (Na_2_SO_4_), and n-hexane (HPLC grade) were obtained from Merck (Darmstadt, Germany). Tocol (2-methyl-2-(4,8,12-trimethyl-tridecyl) chroman-6-ol) was obtained from Matreya Inc. (State College, PA, USA). Vitamin E standards were from Calbiochem (La Jolla, CA, USA). Fatty acid methyl ester standard mixture (FAME) Supelco 37 was obtained from Supelco (Bellefonte, PA, USA). Water was purified in a Milli-Q system (Millipore, Bedford, MA, USA).

### 4.2. Sample Collection

**Figure 1:**
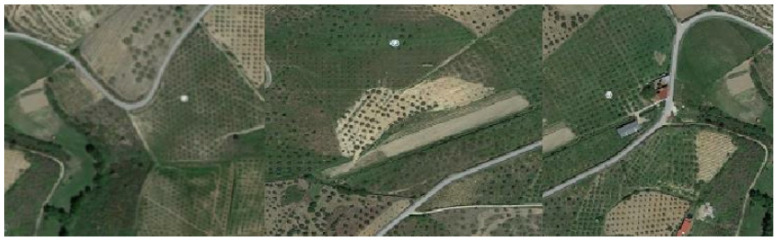
Photos of the harvesting local (aerial view obtained from Google maps according to the respective coordinates). Cultivar (from left to right): Madural, Verdeal, and Cobrançosa.

Olive samples, with origin localization of the olive grove of Valpaços, in Santa Maria de Emeres village, were collected during May. Two types of olive samples were considered, leaves and “*mamões*” (leaf sprouts), from three biological olive cultivars, i.e., Madural, Verdeal, and Cobrançosa. The coordinates in WGS84 for Madural are Lat: 41.540399, Long: −7.390831; for Verdeal, Lat: 41.540463, Long: −7.396549; and for Cobrançosa, Lat: 41.540290, Long: −7.394650 (Figure 1). This area incorporated a total production territory of 8.9 ha, with 1.4 ha of Madural, 1.8 ha of Verdeal, and 5.7 ha of Cobrançosa cultivars. After soil mobilization, and even after a cover based on clover and others was planted, we applied a calcium sulfate complex (Bordeaux mixture without copper) and Sprintplus (algae-based) and applied organic matter with soil correctives according to the analysis. Therefore, no pesticides or herbicides are used to treat the plants, and all products are properly certified for application in organic production or under biological conditions. The number of olive trees per unit of area, on average, is approximately 350 trees per 6 × 6 m^2^, but in the case of the terraces, they are closer. At the start of planting and production, there was financial support for a project, with Ref. Young Farmer: PDR-Rural Development Program, 2012, to initiate the plantation of 3 ha of olive groves in 2018.

### 4.3. Sample Preparation

The preparation of olive leaf samples followed the same procedure used for the analysis of Cydonia oblonga samples since the studied parameters were similar [39]. Thus, olive leaf samples were grounded in a mill (GM200 GrindoMix, Retsch) before organic analysis. For inorganic evaluation, 0.2 g of the dry leaf sample was digested using microwaves (MW) within a closed system at 170 °C using 1 mL of HNO_3_, 2 mL of H_2_O_2_, and 1 mL of H_2_O. After cooling, the vessel contents were transferred to volumetric flasks, and the volume was made up of 25 mL of deionized water.

### 4.4. Inorganic Elements Quantification

The mineral concentration of *O. europaea* L. was obtained by inductively coupled plasma mass spectroscopy (ICP-MS) on a Thermo ICP-MS X Series equipped with a Burgener nebulizer. The system functions with a plasma power at 1400 W, an argon flux of 13 mL/min, an auxiliary gas flux of 1 mL/min, and a sample flux of ~1 mL/min. The tuning procedure was daily performed using a multielement solution (6 Li, In, Ce, U, 10 µg/L each), and the response for oxides (^140^Ce^16^O/^140^Ce ratio) did not exceed 2%. External calibration was performed using multielement standard solution in 1% nitric acid (*v*/*v*) at the following element concentration levels: 0, 0.2, 0.4, 1.0, 2.0, 5.0, 10, 50, and 100 µg/L for minor constituents; and 0, 0.02, 0.04, 0.1, 0.2, 0.5, 1,5, and 10 mg/l for major elements. The internal standard (10.0 µg/L 115 In) was added online. Each analysis was carried out in triplicate readings per solution sample.

### 4.5. Statistical Analysis

Statistical analysis was performed using IBM SPSS Statistics (v. 26 for Windows, IBM Corp., Armonk, 241 NY, USA). The evaluation of statistical significance was determined by ANOVA and Tukey’s HSD to assess significant differences between samples at a 5% significance level.

## 5. Conclusions

The inorganic analysis of the olive leaves of Madural, Verdeal, and Cobrançosa cultivars allowed the proposal of their OLE as a viable and affordable source of mineral substrates to address disorders related to essential elements such as Na, K, Mg, Ca, Mn, Fe, and Cu deficiencies. Deficiencies in minerals such as Ca, Fe, and Mg have been a public health concern, and current advances in food-fortifying techniques as well as in treatment and diagnosis with ion-nanoparticles (ion-NPs) are of utmost importance for world health institutions and governments. Therefore, ion-NPs as a novel therapy targeting mineral and vitamin disorders demand finding a suitable substrate for extracting quality amounts of mineral. In particular, the following can be suggested: (a) Cobrançosa’s leaf sprouts to address hyponatremia; (b) Verdeal’s and Madural’s leaf sprouts for hypokalemia; (c) Verdeal’s elementary leaves and Madural’s leaf sprouts for hypomagnesemia; (d) Cobrançosa’s and Verdeal’s elementary leaves for hypocalcemia; (e) Verdeal’s elementary leaves for Mn deficiency or Madural’s elementary leaves to avoid Mn excess; (f) Verdeal’s leaf sprouts for hypocupremia; and finally (g) the elementary leaves of the three cultivars can be used to address sideropenia. These findings come hand in hand with a previous study about the potential properties of Cydonia oblonga leaves, in which they were demonstrated to have nutritional importance in the context of mineral deficiencies and might serve as cheap potential substrates for food enrichment and supplement manufacture [39]. The physiological influence of enzymes dependent on minerals with regard to neuroinflammatory and neurobehavioral, metabolic, cardiovascular, osteodegenerative, anti-aging, pulmonary, and immunological defense disorders might dictate the importance of further research for designing supplementation based on the nutraceutical potential of OLE of these cultivars predominant in the northern region of Portugal.

## Figures and Tables

**Table 1:** Mineral analysis of leaf samples of *O. europaea*—study made with samples of leaves and leaf sprouts of three varieties at the Valpaços region from the North of Portugal—Madural, Verdeal, and Cobrançosa cultivars of olive tree. ** DRIs (daily recommended intake)—https://nap.nationalacademies.org/read/11537/chapter/59#536 (Accessed on 14 November 2022).

	Cultivar	Units	Madural		Verdeal		Cobrançosa		**DRIs—Male** (31–50 y.o.) **
Elements			Elementary Leaves	Leaf Sprouts (*mamões*)	Elementary Leaves	Leaf Sprouts (*mamões*)	Elementary Leaves	Leaf Sprouts (*mamões*)	
**Al**		mg/Kg	81 ± 0.05	63 ± 0.05	83 ± 0.05	52 ± 0.05	83 ± 0.05	57 ± 0.05	N/A
**As**		mg/Kg	<0.25	<0.25	<0.25	<0.25	<0.25	<0.25	N/A
**B**		mg/Kg	25 ± 0.05	25 ± 0.05	19 ± 0.05	20 ± 0.05	16 ± 0.05	17 ± 0.05	N/A
**Ba**		mg/Kg	11 ± 0.05	19 ± 0.05	10 ± 0.05	5.5 ± 0.03	18 ± 0.05	15 ± 0.05	N/A
**Ca**		g/Kg	8.04 ± 0.03	8.59 ± 0.03	15.29 ± 0.05	6.73 ± 0.03	11.54 ± 0.05	9.10 ± 0.03	1.00 g/d
**Cd**		mg/Kg	0.015 ± 0.03	0.015 ± 0.03	0.014 ± 0.03	0.013 ± 0.03	0.016 ± 0.03	0.014 ± 0.03	N/A
**Co**		mg/Kg	0.82 ± 0.03	0.22 ± 0.03	0.10 ± 0.03	0.19 ± 0.03	0.081 ± 0.03	0.092 ± 0.03	N/A
**Cr**		mg/Kg	0.36 ± 0.03	0.32 ± 0.03	0.31 ± 0.03	0.30 ± 0.03	0.34 ± 0.03	0.31 ± 0.03	35 mg/d
**Cu**		mg/Kg	7.4 ± 0.03	7.3 ± 0.03	5.1 ± 0.03	11 ± 0.05	4.9 ± 0.03	9 ± 0.03	0.90 mg/d
**Fe**		mg/Kg	31 ± 0.05	22 ± 0.05	28 ± 0.05	24 ± 0.05	25 ± 0.05	26 ± 0.05	8 mg/d
**K**		g/Kg	11.53 ± 0.05	12.89 ± 0.05	9.33 ± 0.03	13.12 ± 0.05	7.38 ± 0.03	9.61 ± 0.03	3.40 g/d
**Li**		mg/Kg	0.067 ± 0.03	0.073 ± 0.03	0.066 ± 0.03	0.062 ± 0.03	0.070 ± 0.03	0.062 ± 0.03	N/A
**Mg**		g/Kg	1.02 ± 0.03	1.20 ± 0.03	1.58 ± 0.03	0.73 ± 0.03	0.88 ± 0.03	0.74 ± 0.03	0.42 g/d
**Mn**		mg/Kg	39 ± 0.05	46 ± 0.05	70 ± 0.05	48 ± 0.05	56 ± 0.05	41 ± 0.05	2.3 mg/d
**Mo**		mg/Kg	<0.25	<0.25	<0.25	<0.25	<0.25	<0.25	0.045 mg/d
**Na**		g/Kg	0.053 ± 0.03	0.15 ± 0.03	0.035 ± 0.03	0.10 ± 0.03	0.12 ± 0.03	0.32 ± 0.03	1.5 g/d
**Ni**		mg/Kg	2.6 ± 0.03	3.2 ± 0.03	0.5 ± 0.03	2.0 ± 0.03	4.3 ± 0.03	4.7 ± 0.03	N/A
**P**		g/Kg	3.11 ± 0.03	2.05 ± 0.03	1.96 ± 0.03	2.10 ± 0.03	1.71 ± 0.03	1.72 ± 0.03	0.70 g/d
**Pb**		mg/Kg	0.14 ± 0.03	0.091 ± 0.03	0.12 ± 0.03	0.11 ± 0.03	0.52 ± 0.03	0.15 ± 0.03	N/A
**Rb**		mg/Kg	305 ± 0.05	522 ± 0.05	173 ± 0.05	419 ± 0.05	101 ± 0.05	215 ± 0.05	N/A
**Sb**		mg/Kg	0.020 ± 0.03	0.017 ± 0.03	0.016 ± 0.03	0.015 ± 0.03	0.027 ± 0.03	0.014 ± 0.03	0.06 mg/d
**Se**		mg/Kg	<0.375	<0.375	<0.375	<0.375	<0.375	<0.375	0.055 mg/d
**Si**		mg/Kg	355 ± 0.05	254 ± 0.05	386 ± 0.05	244 ± 0.05	399 ± 0.05	342 ± 0.05	N/A
**Sn**		mg/Kg	<0.375	<0.375	<0.375	<0.375	<0.375	<0.375	N/A
**Sr**		mg/Kg	39 ± 0.05	70 ± 0.05	54 ± 0.05	9 ± 0.03	71 ± 0.05	42 ± 0.05	14.0 mg/d
**Zn**		mg/Kg	62 ± 0.05	20 ± 0.05	21 ± 0.05	28 ± 0.05	15 ± 0.05	18 ± 0.05	11.0 mg/d

## Data Availability

The data presented in this study are available within this article.
